# 

**Published:** 1890-12

**Authors:** 


### CONTENTS:

ARTICLE I.—How to introduce a Gutta-Percha Filling. By Dr. Merit Wells. - - - - - - 337

" II.—Stomatitis.—Diarrhœa in Infants. By H. A. Hare, M. D.. - - - - - - 362

" III.—A Note Concerning the Action of Chloroform. By Dudley Wilmot Buxton, M. D. - - - 369

" IV.—Resuscitation of the Apparently Drowned. - - 379

### EDITORIAL.

Wonders of Surgery. - - - - - - - - 381

### BIBLIOGRAPHICAL.

Descriptive Anatomy of the Human Teeth. - - - - 383

The Dental Laboratory. - - - - - - - 384

Transactions of the American Dental Association. - - - 384

